# *GJB2* Mutation Spectrum and Genotype-Phenotype Correlation in 1067 Han Chinese Subjects with Non-Syndromic Hearing Loss

**DOI:** 10.1371/journal.pone.0128691

**Published:** 2015-06-04

**Authors:** Jing Zheng, Zhengbiao Ying, Zhaoyang Cai, Dongmei Sun, Zheyun He, Yinglong Gao, Ting Zhang, Yi Zhu, Ye Chen, Min-Xin Guan

**Affiliations:** 1 Institute of Genetics, School of Medicine, Zhejiang University, Hangzhou, China; 2 Department of Otolaryngology, Wenling People’s Hospital, Wenzhou Medical University, Taizhou, China; 3 Department of Laboratory Medicine, Jinhua Municipal Central Hospital, Jinhua, China; 4 Attardi Institute of Mitochondrial Biomedicine, School of Laboratory Medicine and Life Sciences, Wenzhou Medical University, Wenzhou, China; 5 Department of Otolaryngology, the First Affiliated Hospital, Wenzhou Medical University, Wenzhou, China; 6 Collaborative Innovation Center for Diagnosis and Treatment of Infectious Diseases, Zhejiang University, Hangzhou, China; Odense University hospital, DENMARK

## Abstract

Mutations in Gap Junction Beta 2 (*GJB2*) have been reported to be a major cause of non-syndromic hearing loss in many populations worldwide. The spectrums and frequencies of *GJB2* variants vary substantially among different ethnic groups, and the genotypes among these populations remain poorly understood. In the present study, we carried out a systematic and extended mutational screening of *GJB2* gene in 1067 Han Chinese subjects with non-syndromic hearing loss, and the resultant *GJB2* variants were evaluated by phylogenetic, structural and bioinformatic analysis. A total of 25 (23 known and 2 novel) *GJB2* variants were identified, including 6 frameshift mutations, 1 nonsense mutation, 16 missense mutations and 2 silent mutations. In this cohort, c.235delC is the most frequently observed pathogenic mutation. The phylogenetic, structural and bioinformatic analysis showed that 2 novel variants c.127G>T (p.V43L), c.293G>C (p.R98P) and 2 known variants c. 107T>C (p.L36P) and c.187G>T (p.V63L) are localized at highly conserved amino acids. In addition, these 4 mutations are absent in 203 healthy individuals, therefore, they are probably the most likely candidate pathogenic mutations. In addition, 66 (24 novel and 42 known) genotypes were identified, including 6 homozygotes, 20 compound heterozygotes, 18 single heterozygotes, 21 genotypes harboring only polymorphism(s) and the wild type genotype. Among these, 153 (14.34%) subjects were homozygous for pathogenic mutations, 63 (5.91%) were compound heterozygotes, and 157 (14.71%) carried single heterozygous mutation. Furthermore, 65.28% (141/216) of these cases with two pathogenic mutations exhibited profound hearing loss. These data suggested that mutations in *GJB2* gene are responsible for approximately 34.96% of non-syndromic hearing loss in Han Chinese population from Zhejiang Province in eastern China. In addition, our results also strongly supported the idea that other factors such as alterations in regulatory regions, additional genes, and environmental factors may contribute to the clinical manifestation of deafness.

## Introduction

Hearing loss is one of the most common sensory defects, affecting one in 700–1000 newborns[1,2]. It can be classified as syndromic hearing loss (hearing loss with other symptoms such as diabetes), and non-syndromic hearing loss (hearing loss is the only obvious clinical phenotype). Approximately 50% of all cases of hearing loss have a genetic etiology or predisposition with autosomal dominant, autosomal recessive, X-linked or maternal patterns of inheritance[3,4]. To data, over 140 loci have been mapped for non-syndromic hearing loss, and 82 deafness-causing genes have been identified (The Hereditary Hearing loss Homepage. http://hereditaryhearingloss.org).

Mutations in the *GJB2* gene, which encodes the connexin 26 (Cx26), are a major cause of non-syndromic hearing loss in many populations worldwide[5]. More than 150 different *GJB2* variants have been identified, including missense, nonsense and frameshift mutation (The Connexin-deafness Homepage. http://davinci.crg.es/deafness). The spectrums of *GJB2* mutations vary among different ethnic groups[5,6]. Of these variants, the c.35delG is the most prevalent *GJB2*-deafness-causing mutation in European populations, while c.235delC and c.167delT are the most frequent variants in Eastern Asian populations and Ashkenazi Jewish families, respectively[5,7–10]. In addition, the prevalence of *GJB2* mutations differs among various populations, which varied from 7.9% to 44.1% among Caucasian cohorts and 4.5% to 34.7% in several Asian populations[5,6,11–17]. However, the spectrum and frequency of *GJB2* mutations in the Chinese population are still poorly understood, and the prevalence of various *GJB2* genotypes among different Chinese populations is less studied. In addition, it is anticipated that there are additional *GJB2* mutation(s) associated with hearing loss among Chinese population.

In the present study, a systematic and extended mutational screening of *GJB2* gene was carried out in a cohort of 1067 Han Chinese hearing-impaired subjects from Zhejiang Province, in Eastern China. We found 25 variants in this large cohort including missense mutation, nonsense mutation and frameshift mutation, which could be attributed to 66 genotypes. By phylogenetic analysis, structure-function relation and allelic frequency of these variants in the 203 Han Chinese healthy individuals from the same region, 4 putative pathogenic mutations were identified.

## Subjects and Methods

### Subjects and audiological examinations

A total of 1067 genetically unrelated hearing-impaired Chinese subjects were recruited from Zhejiang Province in Eastern China. The subjects were composed of 509 students from Schools for Deaf Children and Hearing and Speech Rehabilitation Centers, and 558 patients from the ENT Clinics of Zhejiang Province. A comprehensive history and physical examination were performed on these participants, so as to identify any syndromic findings, the history of the use of aminoglycosides, environmental and genetic triggers related to the hearing loss. Age-appropriate audiological evaluation was carried out as previous report [18]. Briefly, the pure-tone audiometry (PTA) was calculated from average of the audiometric thresholds at 500, 1000, 2000, 4000 and 8000 Hz. The severity of hearing loss was then classified into five grades: normal (<26 dB), mild (between 26 and 40 dB), moderate (between 41 and 70 dB), severe (between 71 and 90 dB), and profound (>90 dB). Auditory brainstem response (ABR), immittance testing and distortion product otoacoustic emissions (DPOAE) were also performed. The control DNA samples were obtained from 203 healthy Han Chinese subjects in the same region of China. This study has been approved by the Ethics Committees of both Zhejiang University and Wenzhou Medical University, and written informed consent was obtained from all participants.

### Mutational analysis of GJB2 gene

Genomic DNAs were isolated from the blood samples of all participants using Universal Genomic DNA Extraction Kit Ver.3.0 (TaKaRa, Dalian, China). The DNA fragments spanning the entire coding region of *GJB2* gene were amplified by PCR using the oligodeoxynucleotides: forward-5’TATGACACTCCCCAGCACAG3’ and reverse-5’GGGCAATGCTTAAACTGGC3’. Then the purified *GJB2* fragments were analyzed by direct sequencing as detailed previously[19]. Subsequently, the mutations were identified by comparing the resultant sequence with wild type *GJB2* sequence (GenBank accession No: NM_004004.5). The sequence of each amplicon was confirmed by sequencing in both directions.

### Phylogenetic analysis

A total of 23 species Cx26 amino acid sequences were used in the inter-species analysis (S2 Table). The conservation index (CI) was defined as the percentage of species with amino acid residues identical to that of human at that position. Alignments and analysis were performed using Clustal Omega (http://www.ebi.ac.uk/Tools/msa/clustalo/).

### Bioinformatic analysis

In order to predict the effect of the putative *GJB2* mutation on protein function, the pathogenic potential of these missense mutations was evaluated by PolyPhen-2 (http://genetics.bwh.harvard.edu/pph2/index.shtml) and SIFT (http://sift.jcvi.org/) programs.

## Results

### Study samples

The study samples were Han Chinese recruited from Schools of Deaf Children and ENT clinics of Zhejiang Province. Subjects who had a history of exposure to aminoglycoside antibiotics, or other clearly identifiable cause(s) of hearing loss (such as bacterial meningitis, labyrinthitis, temporal bone fracture involving the cochlea, active chronic otitis media, cholesteatoma), or syndromic deafness were excluded from this study. A total of 1067 subjects (609 males and 458 females), who exhibited sensorineural hearing loss as the sole symptom, were analyzed. The age of subjects ranged from 1 year old to 41 years old, with a median age of 17 years. The age-at-onset of the hearing loss in these subjects ranged from congenital to 41 years old, with a median age of 8 years. Audiological studies showed that there was a wide range of different degrees of hearing loss in these subjects: 485 subjects exhibited profound hearing loss, 403 patients had severe hearing loss, 145 individuals suffered moderate hearing loss, and 34 cases with mild hearing loss. In addition, 203 Han Chinese subjects (112 males and 91 females) recruited from the same region, exhibited normal hearing and didn’t have a family history of hearing loss. The age of these participants ranged from 8 years old to 27 years old, with a median age of 16 years (S1 Table).

### Mutation analysis of *GJB2* gene

DNA fragments spanning the entire coding region of *GJB2* gene were PCR-amplified from genomic DNA samples of 1067 hearing-impaired and 203 control subjects, and each fragment was sequenced. As shown in Table 1, 25 (2 novel and 23 known) nucleotide changes in *GJB2* gene were identified when comparing the resultant sequence with the wild type sequence (GenBank accession NM_004004.5). Among these mutations, 2 novel variants (p.V43L and p.R98P) were identified in heterozygous state. When looking at the allelic frequency of *GJB2* gene in healthy individuals, we found 3 variants (p.V27I, p.V37I and p.E114G) in 203 Chinese controls with the frequencies of 22.91%, 4.93% and 15.02%, respectively. Furthermore, the missense mutations were evaluated with phylogenetic analysis by comparing the human *GJB2*-encoding connexin-26 (Cx26) amino acid residues with other 22 species in data bank. As shown in Table 1, all the 16 missense mutations observed in our study changed amino acids which are well conserved in evolution with the conservation index (CI) ranged from 39.13% to 100%. In particular, CI of 13 variants were >90%, CI of other 2 variants including p.G4D and p.R98P were between 80% and 50%, and CI for p.T123N variant was 39.13%. In addition, localization of these mutations in the secondary structure of Cx26 protein was exhibited in S1 Fig The variants p.V43L, p.V63L and p.G160S reside at the extracellular loops of Cx26, the variants p.V27I, p.I30V, p.L36P, p.V37I, p.R143W, p.E147K and p.I203T occur in transmembrane domains, and the variants p.G4D, p.G12V, p.V95M, p.R98P, p.E114G and p.T123N are located at amino termini and intracellular loop of the protein. Moreover, both the PolyPhen-2 and SIFT programs showed that 9 of these missense mutations were damaging (Table 1).

**Table 1:** Variants in the *GJB2* gene among 1067 Han Chinese subjects with hearing loss.

Nucleotide Change	Effect on Protein	dbSNP ID	Conservation Index (%)^a^	Allele Frequency in Affected Subjects (%, 2134 allele)	Allele Frequency in Controls (%, 406 allele)	PolyPhen-2	SIFT	Characterization of Variant
**Frameshift mutations** (Deletions and insertions)								
c.35insG	G12GfsX36		-	0.09	0			Pathogenic
c.35delG	G12VfsX2	rs80338939		0.09	0			Pathogenic
c.176_191del16	G59AfsX18		-	0.75	0			Pathogenic
c.235delC	L79CfsX3	rs80338943	-	13.96	0			Pathogenic
c.299_300delAT	H100RfsX14	rs111033204	-	2.25	0			Pathogenic
c.512_513insAACG	A172EfsX39		-	0.52	0			Pathogenic
**Nonsense mutation**								
c.139G>T	p.E47X	rs104894398	100	0.09	0			Pathogenic
**Missense mutations**								
c.11G>A	p.G4D	rs111033222	56.52	0.33	0	Benign	Tolerated	Polymorphism
c.35G>T	p.G12V	rs1801002	100	0.05	0	Damaging	Damaging	Pathogenic
c.79G>A	p.V27I	rs2274084	100	25.21	22.91	Damaging	Tolerated	Polymorphism
c.88A>G	p.I30V	rs374625633	100	0.14	0	Benign	Tolerated	Polymorphism
c.107T>C	p.L36P		100	0.05	0	Damaging	Damaging	Putative pathogenic
c.109G>A	p.V37I	rs72474224	100	8.86	4.93	Damaging	Tolerated	Pathogenic
c.127G>T^b^	p.V43L		100	0.05	0	Damaging	Damaging	Putative pathogenic
c.187G>T	p.V63L		100	0.05	0	Damaging	Damaging	Putative pathogenic
c.283G>A	p.V95M	rs111033299	91.30	0.09	0	Damaging	Damaging	Pathogenic
c.293G>C^b^	p.R98P		78.26	0.05	0	Damaging	Damaging	Putative pathogenic
c.341A>G	p.E114G	rs2274083	91.30	19.07	15.02	Benign	Tolerated	Polymorphism
c.368C>A	p.T123N	rs111033188	39.13	0.23	0	Benign	Tolerated	Polymorphism
c.427C>T	p.R143W	rs80338948	100	0.09	0	Damaging	Damaging	Pathogenic
c.439G>A	p.E147K		100	0.09	0	Damaging	Damaging	Pathogenic
c.478G>A	p.G160S	rs34988750	100	0.33	0	Damaging	Tolerated	Polymorphism
c.608T>C	p.I203T	rs76838169	95.65	0.14	0	Damaging	Damaging	Polymorphism
**Silent variants**								
c.81C>T	p.V27		100	0.05	0			Polymorphism
c.444C>T	p.A148		100	0.05	0			Polymorphism

^a^ The conservation index (CI) was calculated by comparing the human amino acid variants with other 22 species (See S1 Table). The CI was then defined as the percentage of species from the list of 23 different species that have the wild-type nucleotide at that position.

^b^ The novel variants.

The p.G4D, p.V27I, p.E114G and p.T123N variants were considered as polymorphisms to comply with the standard of whose frequency >5% in controls or CIs<60%. In addition, the p.I30V, p.G160S and p.I203T variants were also classified as benign since they were reported in controls in other populations[6,13,20,21]. On the other hand, the frequency of p.V37I variant in hearing-impaired subjects was much higher than in our control cohort. Furthermore, this variant has been reported to be associated with mild hearing loss among Chinese and Japanese populations[22]. Taken together, we identified 9 *GJB2* variants in this large cohort of Han Chinese population to be pathogenic, including the 5 known deafness-associated mutations (p.G12V, p.V37I, p.V95M, p.R143W and p.E147K). The allelic frequency of pathogenic mutations in *GJB2* gene among this Chinese cohort was 27.13%. In particular, c.235delC was the most frequent mutation with the allelic frequency of 13.96%, followed by c.299_300delAT (2.25%).

### Genotypes and phenotypes of *GJB2*

As shown in S3 Table, a total of 66 (24 novel and 42 known) genotypes of *GJB2* was observed in the studied cohort of 1067 hearing-impaired subjects, including 44 (16 novel and 28 known) genotypes carrying at least one of pathogenic mutation(s), 21 (8 novel and 13 known) genotypes harboring only polymorphism(s) and a genotype lacking any copy of variants. In this study, we found 34.96% (373/1067) of the hearing-impaired cases carrying at least one pathogenic mutation, among which 153 (14.34%) were homozygous for pathogenic mutations and 63 (5.91%) were compound heterozygotes, while the other 157 (14.71%) subjects only had one heterozygous mutation. In particular, c.235delC homozygote was the most common pathogenic genotype, accounting for 70.59% (108/153) of the homozygotes group, and 10.12% of the entire subjects. Furthermore, the [c.235delC]/[c.299_300delAT] was found to be the most frequent genotype with a frequency of 30.16% (19/63) in compound homozygotes group. Among the 216 subjects with biallelic pathogenic mutations (Table 2), 65.28% (141/164) of these cases exhibited profound hearing loss.

**Table 2:** Genotypes and phenotypes of *GJB2* in the 216 hearing-impaired subjects with two pathogenic mutations.

Genotypes	Severity of Hearing Loss _(PTA0.5-8k Hz)_ ^a^				Number
	Mild	Moderate	Severe	Profound	
**[c.235delC]** ^b^ **/ [c.235delC]**	1	2	23	82	108
**[c.235delC**; c.478G>A**] / [c.235delC]**	0	0	0	1	1
**[c.235delC**; c.478G>A**] / [c.235delC**; c.478G>A**]**	0	0	0	1	1
**[c.299_300delAT] / [c.299_300delAT]**	0	1	0	5	6
**[c.109G>A] / [c.109G>A]**	5	13	9	7	34
[c.79G>A;**c.109G>A**;c.341A>G] **/ [c.109G>A]**	0	2	0	1	3
**[c.235delC] / [c.35insG]**	0	0	0	2	2
**[c.235delC] / [c.35delG]**	0	0	0	1	1
**[c.235delC] / [c.35G>T**;c.79G>A; c.341A>G]	0	0	1	0	1
**[c.235delC] / [c.127G>T**;c.79G>A; c.341A>G]	0	0	0	1	1
**[c.235delC] / [c.139G>T]**	0	0	0	1	1
**[c.235delC] / [c.176_191del16]**	0	1	2	4	7
**[c.235delC] / [c.299_300delAT]**	0	0	4	15	19
**[c.235delC**;79G>A] **/ [c.299_300delAT]**	0	0	0	1	1
**[c.235delC**;79G>A] **/ [c.439G>A]**	0	1	0	1	2
**[c.235delC] / [c.512_513insAACG]**	0	2	1	3	6
**[c.299_300delAT] / [c.139G>T]**	0	0	0	1	1
**[c.299_300delAT] / [c.176_191del16]**	0	0	0	5	5
**[c.299_300delAT] / [c.512_513insAACG]**	0	0	0	1	1
**[c.235delC] / [c.109G>A]**	2	1	1	1	5
**[c.176_191del16] / [c.109G>A]**	0	0	0	2	2
**[c.283G>A] / [c.109G>A]**	0	0	0	1	1
**[c.283G>A; c.79G>A; c.341A>G] / [c.109G>A]**	0	0	1	0	1
**[c.293G>C] / [c.109G>A]**	0	0	0	1	1
**[c.299_300delAT] / [c.109G>A]**	0	1	0	2	3
**[c.427C>T] / [c.109G>A]**	0	0	1	1	2
**Total**	8	24	43	141	216

^a^ PTA pure-tone audiometry.

^b^ The pathogenic mutations were in bold.

Among the 18 genotypes of single heterozygotes, 16 (4 novel and 12 known) genotypes carried pathogenic mutations (c.35delG, c.176_191del16, c.235delC, c.299_300delAT, c.512_513insAACG, p.V37I, p.V95M and p.R143W) and 2 novel genotypes harbored the putative deafness-associated mutations (p.L36P and p.V63L). In addition to the genotypes contained pathogenic mutation(s) mentioned above, there were 22 genotypes did not carry any deafness-associated mutation(s), involving 65.04% (694/1067) cases of this cohort. Of these nonpathogenic genotypes, 21 genotypes were found only harboring polymorphisms including 2 genotypes with silent variants (c.81C>T, p.V27 and c.444C>T, p.A148) and 19 genotypes carrying missense variants (p.G4D, p.V27I, p.I30V, p.E114G, p.T123N, p.G160S and p.I203T).

### Clinical and genetic characterization of hearing-impaired subjects carrying the 4 putative *GJB2* mutations

A genotype-phenotype correlation analysis was performed among 4 probands carrying the 4 putative deafness-associated *GJB2* mutations. The comprehensive history and physical examination including audiological assessment indicated that hearing impairment was the sole clinical phenotype in these subjects, and no other clinical abnormalities was observed in members of their families such as diabetes, muscular diseases, visual loss and neurological disorders. All the 4 subjects did not have a history of exposure to neither aminoglycosides nor other ototoxic drugs. Moreover, there was no evidence that the 4 cases had encountered any other external cause to be responsible for hearing loss. Audiological assessments indicated that they all exhibited profound hearing loss at ages younger than 2 years old, with flat-shaped pattern of audiometric configuration.

**Fig 1:**
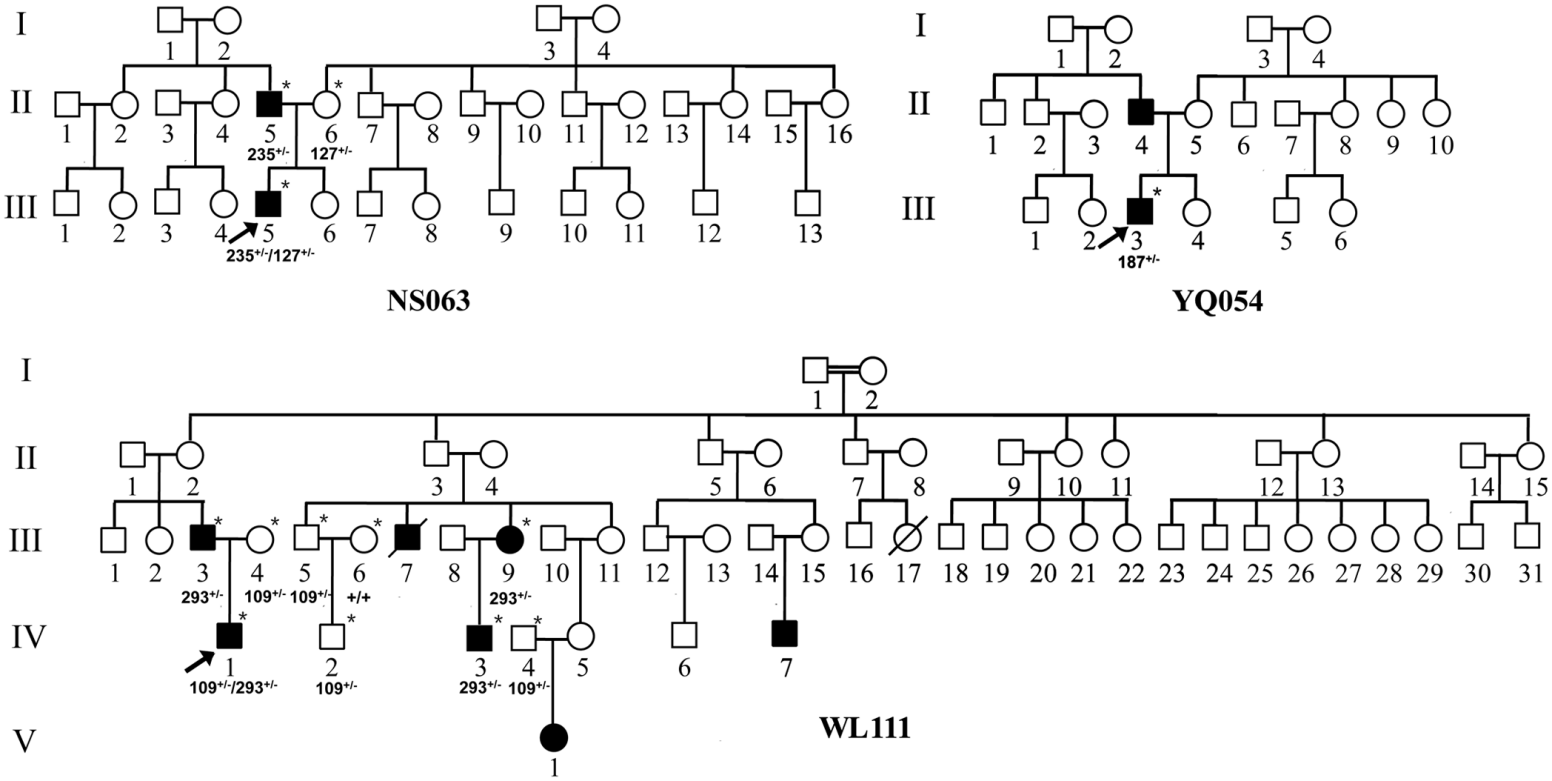
Three Han Chinese pedigrees with hearing loss carrying the *GJB2* putative mutations. Affected individuals are indicated by filled symbols. An arrow denotes probands. The interviewed and sequenced individuals are marked by asterisks. +/- denotes heterozygote; +/+ denotes wild type.

As illustrated in Table 3 and Fig 1, 2 of the 4 subjects had a family history of hearing loss. Proband NS063-III-5, whose genotype was in a heterozygous state of c.127G>T (p.V43L) and c.235delC mutations, suffered profound congenital hearing loss. Among the other family members, only his father exhibited moderate hearing defect (45 dB at right ear, 42 dB at left ear; sloping-shaped pattern). In addition, the proband WL111-IV-1, bearing the [c.293G>C]**/**[c.109G>A] genotype, showed profound congenital hearing loss. Among this pedigree, the c.293G>C (p.R98P) variant was also detected in III-3, III-9 and IV-3 in heterozygous state. Audiological examinations showed mild hearing loss in III-3 with sloping-shaped pattern, and the III-9 and IV-3 exhibited profound congenital hearing loss.

**Table 3:** Summary of clinical and molecular data for 4 Han Chinese subjects carrying the putative deafness-associated mutations in *GJB2* gene.

Genotype	Proband	Gender ^a^	Audiometric Configuration	Age-at-onset (Years)	PTA^b^(dB)^c^ Right Ear	PTA(dB) Left Ear	Level of Hearing Loss	Family History of Hearing Loss
**[c.107T>C]** ^**d**^ **/** [c.79G>A; c.341A>G]	NS110	F	Flat	<1	114	108	Profound	No
**[c.235delC**;c.79G>A; c.341A>G**] / [c.127G>T]**	NS063	M	Flat	<1	115	113	Profound	Yes
**[c.187G>T] /** [c.79G>A; c.341A>G]	YQ054	F	Flat	2	109	110	Profound	No
**[c.293G>C] / [c.109G>A]**	WL111	F	Flat	<1	108	112	Profound	Yes

^a^ F female, M male.

^b^ PTA pure-tone audiometry.

^c^ dB decibel.

^d^ The (putative) pathogenic mutations were in bold.

## Discussion

*GJB2* is the most common causative gene for hereditary hearing loss in many populations worldwide, and most of the *GJB2* sequence variations described to date were localized in the coding region (The Connexin-deafness Homepage. http://davinci.crg.es/deafness). In fact, the *GJB2* mutation spectrums and frequencies vary among different ethnic population[5,6]. In the present study, we analyzed the spectrum and frequency of *GJB2* variants in 1067 Chinese non-syndromic hearing loss subjects from Zhejiang Province in eastern China, and investigated the clinical and genetic characterization of patients with putative *GJB2* mutations. A total of 25 different nucleotide changes in the *GJB2* gene were identified in this cohort, 34.96% of subjects in the cohort harbored *GJB2* pathogenic mutations, which can be classified into 44 genotypes, including homozygotes, compound heterozygotes and single heterozygotes (only one mutant allele). These results indicate that *GJB2* mutations are the important causes of hearing loss in hearing-impairment Han Chinese population.

In the present study, we revealed a high prevalence (27.13%) of *GJB2* mutations in Chinese hearing loss patients, which is compatible with other reports[13,23]. Among these variants, c.235delC mutation was observed with a frequency of 13.96% in our cohort. The c.235delC mutation in the *GJB2* gene predominantly occurs in people of East Asian, the incidences of the mutation ranged from 6.7% to 22.5% in several other Chinese deaf cohorts according to previous studies, which were 5.3% and 10.9% in two reported Japanese hearing loss cohorts, 1.5% in a cohort from Mongolia, 5.1% and 6.9% in two cohorts of Korean deafness patients[11,14,24–28]. The high frequency of c.235delC mutation in multiple East Asian populations raises the possibility of a common ancestral founder event. Interestingly, c.235delC mutation has not been reported in south Asian populations such as Indian, Pakistanis, and Bangladeshis[5]. Instead, p.W24X, p.W77X and p.Q124X are the prevalent *GJB2* mutations in these populations[5,12,29,30]. Interestingly, the observed racial divergence of South and East Asian is consisted with the studies of mtDNA haplogroup analysis in that predicting country of ancestral origin (http://www.mitomap.org, 2015). It has been well accepted that haplogroup L3 gave rise to two lineages: M and N. In addition, the major haplogroups B and F in East Asian were rising from macrohaplogroup N, and the mitochondrial haplogroups in South Asian were macrohaplogroup M and its derivatives. These results indicate that c.235delC mutation might have arisen after the racial divergence of South and East Asian occurred. It would be necessary to systematically investigate the *GJB2* mutations of remaining Asian countries to precisely elucidate the origin of c.235delC mutation[31]. Of the other *GJB2* deletion/frameshift mutations associated with hearing loss, c.176_191del16 and c.299_300delAT were previously reported in Korean and Japanese hearing loss cohorts[14,25], and c.512_513insAACG mutation has been also detected in Chinese and Japanese deaf patients but in a much lower frequency[13,14].

The c.35delG mutation of *GJB2* was previously reported to be the most prevalent mutation (21%) in a cohort of north Indian population with hearing loss[32]. In particular, among these populations with European ancestry, the c.35delG mutation causes severe to profound hearing loss, accounted for approximately 70% of all recessive mutations of the gene[7]. In fact, the mutations at position 35 are found to be exceptionally low in eastern Asian. Dai et al reported in a large cohort that 12 Chinese patients carried c.35delG mutation; however, the majority of these patients was Uigur from Xinjiang area[13]. In this cohort of Han people from Zhejiang Province in eastern China, the deletion, insertion and G to T mutation at position 35 were respectively detected in 5 unrelated subjects, with all the 3 mutations found in compound heterozygous state. Of the 5 cases, 4 subjects also carried c.235delC mutation, and the other one patient had c.79G>A polymorphism.

Fifteen additional missense mutations (13 known and 2 novel) were detected among these hearing loss patients, including 4 pathogenic mutations, 7 polymorphisms and 4 unknown variants. To identify putative pathogenic mutation, the unknown variants were further evaluated using following criteria: (1) conservation index greater than 75%, (2) absent in the 203 controls, (3) potential structural and functional alterations, predicted by their locations, PolyPhen-2 and SIFT programs, and (4) pedigree analysis if possible. The p.L36P, p.V43L, V63L and p.R98P variants, which occurred at evolutionarily conserved amino acid residues and absent from the control group, were predicted as pathogenic mutations by PolyPhen-2 and SIFT programs. The p.L36P mutation was first described in *trans* with 35delG in a hearing loss patient of African descent[33]. This putative disease-causing mutation locates at the transmembrane domain of Cx26 protein, which is involved in the oligomerization of hexameric connexon hemichannels. The resultant changed protein may lead to the reduction of junctional permeability in cells that is essential for ions and small metabolites exchange. Variants p.V43L and V63L, which reside at the first extracellular loop of Cx26, may result in gap-junction channels dysfunction among adjacent cells. The Val at 43 position changed to Met in Cx26 was previously observed in a hearing loss cohort from Taiwan and considered to be associated with the disease[34]. Here, we report the novel p.V43L variant as a potential deafness-associated mutation in Han Chinese population from Zhejiang province. The p.V63L mutation was first reported in a Taiwan cohort, and then it was also found in other Chinese population with hearing loss[13,34]. However, the pathogenic potential of this variant is difficult to define since it has been reported only in the heterozygous state without accompanying other mutations. Another novel *GJB2* variant we found in this study was the p.R98P mutation which occurred in amino termini and intracellular loop, it is proposed that this mutation may impair the gap junctional intercellular communication in cochlea and finally lead to hair cell dysfunction. More interestingly, we found that the p.R98P variant co-segregates with the phenotype of hearing loss in the WL111 pedigree (Fig 1). But based on the current data, it is not possible to determine whether this variant is a dominant or a recessive mutation. Taking together, these 4 amino acid changes are very likely to be the possible causes of hearing loss. However, the functional impact of these putative causal variants remains speculative, the establishment of recombinant expression systems and animal models would be necessary for further investigation.

The p.V37I variant was originally reported as a polymorphism[35]. Subsequently, a series of studies reported that this variant exists in deafness patients in homozygous or compound heterozygous state among different populations[5,36]. However, its pathogenicity is still controversial. Ethnic differences were observed in the allele frequency of p.V37I variant, as it was not detected in the control subjects from Italy, Spain, Germany, Greece, Israel, Ghana, and Austria in spite of a high prevalence of the mutation in Eastern Asian population such as Chinese and Japanese descent[22]. In addition, the p.V37I mutant protein has normal subcellular localization and membrane expression as the wild type protein[37]. In this study, we found that the V37I allele was identified in 8.86% and 4.93% of the Chinese patient and control alleles, respectively. The p.V37I variant is located at transmembrane domain of Cx26, and the Valine residue at position 37 is highly conserved among different species. Moreover, functional analysis has proved that the coexpression at equimolar levels of wild type and mutant Cx26 proteins (p.V37I) in *Xenopus* oocytes resulted in a dominant-negative effect of the mutation[38]. In general, this variant results in a rather mild phenotype, which is consistent with previous reports that p.V37I missense mutation leads to a less severe hearing loss phenotype than frameshift mutations such as c.35delG and c.235delC mutations[39,40]. Taking into consideration the analysis of clinical, genetical and functional data regarding p.V37I available in the publications, this variant could also be a primary cause that leads to predisposition for hearing loss depending on genetic background and/or environmental factors.

In this report, we found 66 genotypes of *GJB2* in the large cohort of 1067 hearing-impaired subjects including 24 novel genotypes. Totally, 20.25% of the subjects harbored two *GJB2* pathogenic alleles, either homozygotes or compound heterozygotes, and there was a fairly high frequency (14.71%) of single heterozygosity in this cohort. More interestingly, patients with homozygous or compound heterozygous for the same pathogenic mutation exhibit a wide variation in the phenotypic expression of hearing loss. These data strongly supported the hypothesis that other factors may contribute to the clinical manifestation of *GJB2*-related deafness, such as alterations in regulatory region, additional mutations in other genes such as *GJB6* and *GJB3*, and environmental factors[6,41,42]. Accordingly, identification of *GJB2* mutations can yield considerable helpful information to those hearing impaired population and their families. Understanding of the cause of hearing loss will effectively promote genetic counseling and implementation of various intervention strategies such as early rehabilitation and fitting of hearing aids, which is particularly important for infants in affected families. In addition, further studies emphasize on the disease associated factors are very important that would enable us to gain a deeper understanding of the complex molecular pathogenic mechanisms of hearing loss.

## Supporting Information

S1 Fig The schematic topology of Cx26 and the distribution of variants. The Cx26 protein has four transmembrane domains (M1–M4), connected by two extracellular loops (E1 andE2) and a cytoplasmic loop (CL). The NT and CT denote the N- and C-termini of the protein.
(TIF)

S1 Table The characteristics of hearing impaired subjects and healthy control subjects. (DOC)

S2 Table Cx26 amino acid sequence data of 23 species. (DOC)

S3 Table Genotypes of *GJB2* gene in 1067 Han Chinese subjects with hearing loss. (DOCX)
