# Supplementary figures and images for: GJB2 Mutation Spectrum and Genotype-Phenotype Correlation in 1067 Han Chinese Subjects with Non-Syndromic Hearing Loss
Source: PLoS One. 2015 Jun 4;10(6):e0128691. doi: 10.1371/journal.pone.0128691 (PMC4456361; doi:10.1371/journal.pone.0128691)

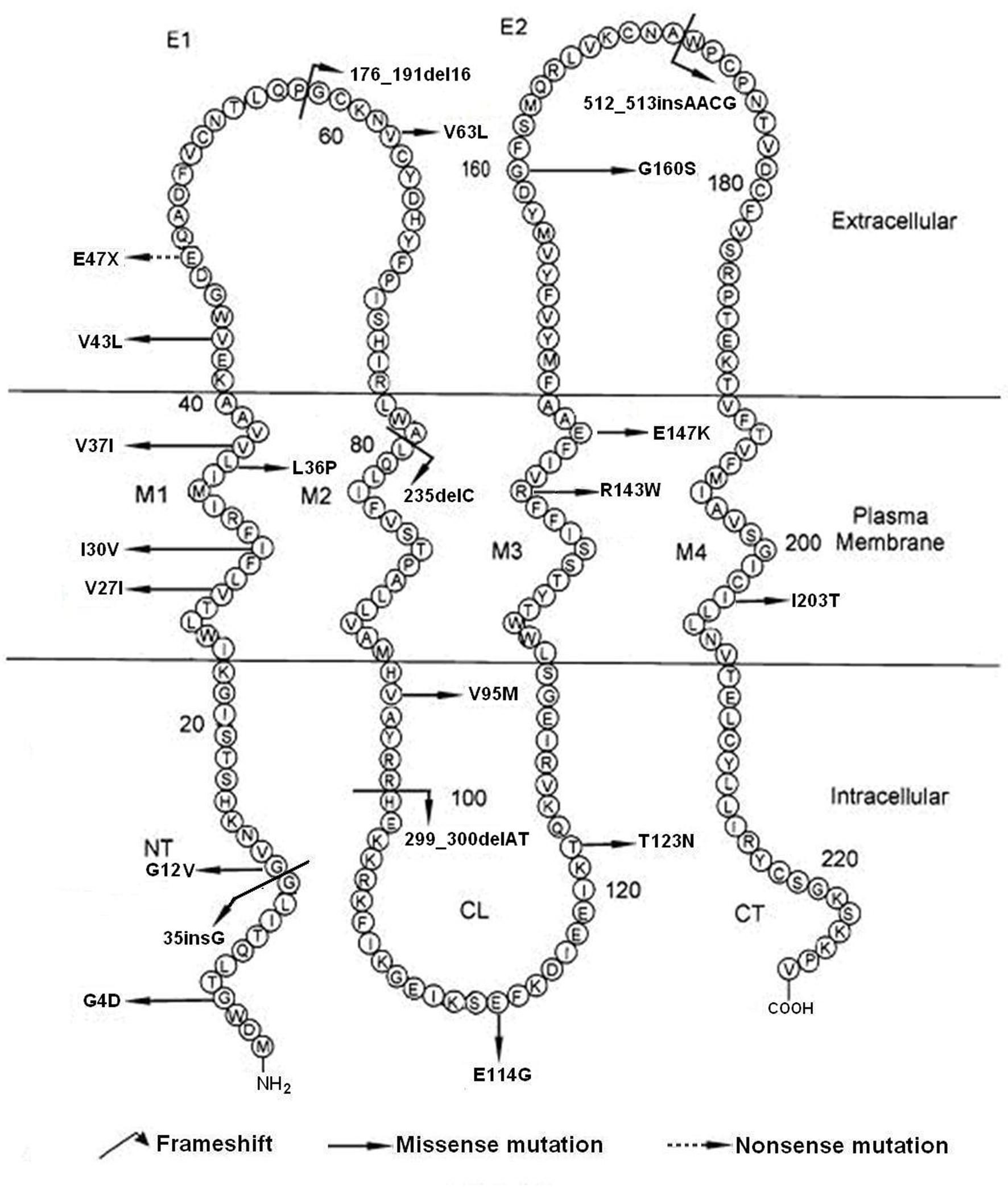

Supplement: S1 Fig — The Cx26 protein has four transmembrane domains (M1–M4), connected by two extracellular loops (E1 andE2) and a cytoplasmic loop (CL). The NT and CT denote the N- and C-termini of the protein. (TIF) [file pone.0128691.s001.tif]
