# Supplementary material for: GJB2 Mutation Spectrum and Genotype-Phenotype Correlation in 1067 Han Chinese Subjects with Non-Syndromic Hearing Loss
Source: PLoS One. 2015 Jun 4;10(6):e0128691. doi: 10.1371/journal.pone.0128691 (PMC4456361; doi:10.1371/journal.pone.0128691)
Supplement: S1 Table — (DOC) [file pone.0128691.s002.doc]

**Table S1.** The characteristics of hearing impaired subjects and healthy control subjects.

| **Variable** | **Hearing impaired subjects** | **Healthy control subjects** |
| --- | --- | --- |
| Number | 1067 | 203 |
| Age median (years) | 17 (1-41) | 16 (8-27) |
| Gender (male/female) | 609/458 | 112/91 |
