# Supplementary material for: GJB2 Mutation Spectrum and Genotype-Phenotype Correlation in 1067 Han Chinese Subjects with Non-Syndromic Hearing Loss
Source: PLoS One. 2015 Jun 4;10(6):e0128691. doi: 10.1371/journal.pone.0128691 (PMC4456361; doi:10.1371/journal.pone.0128691)
Supplement: S2 Table — (DOC) [file pone.0128691.s003.doc]

**Table S2. Cx26 amino acid sequence data of 23 species**.

| **Species name** | **Number (Ensembl)^a^** |
| --- | --- |
| *Homo sapiens* | ENSP00000372299 |
| *Pan troglodytes* | ENSPTRP00000053675 |
| *Gorilla gorilla* | ENSGGOP00000016284 |
| *Callithrix jacchus* | ENSCJAP00000040706 |
| *Otolemur garnettii* | [ENSOGAP00000010449](http://www.ensembl.org/Otolemur_garnettii/Transcript/ProteinSummary?db=core;g=ENSOGAG00000011675;r=scaffold_8608:8184-8864;t=ENSOGAT00000011678) |
| *Macaca mulatta* | [ENSMMUP00000013790](http://www.ensembl.org/Macaca_mulatta/Transcript/ProteinSummary?db=core;g=ENSMMUG00000010522;r=17:1318135-1323484;t=ENSMMUT00000014720) |
| *Pongo pygmaeus* | [ENSPPYP00000005913](http://www.ensembl.org/Pongo_pygmaeus/Transcript/ProteinSummary?db=core;g=ENSPPYG00000005190;r=13:19280971-19281651;t=ENSPPYT00000006145) |
| *Tursiops truncatus* | ENSTTRP00000003504 |
| *Loxodonta africana* | ENSLAFP00000023594 |
| *Dasypus novemcinctus* | ENSDNOP00000007891 |
| *Felis catus* | ENSFCAP00000006458 |
| *Procavia capensis* | ENSPCAP00000007566 |
| *Ochotona princeps* | ENSOPRP00000004933 |
| *Oryctolagus cuniculus* | ENSOCUP00000008752 |
| *Mus musculus* | ENSMUSP00000054343 |
| *Dipodomys ordii* | ENSDORP00000005560 |
| *Rattus norvegicus* | ENSRNOP00000011711 |
| *Microcebus murinus* | [ENSMICP00000002590](http://www.ensembl.org/Microcebus_murinus/Transcript/ProteinSummary?db=core;g=ENSMICG00000002850;r=GeneScaffold_1502:143828-144508;t=ENSMICT00000002844) |
| *Monodelphis domestica* | ENSMODP00000033449 |
| *Ornithorhynchus anatinus* | ENSOANP00000014354 |
| *Bos Taurus* | [ENSBTAP00000023167](http://www.ensembl.org/Bos_taurus/Transcript/ProteinSummary?db=core;g=ENSBTAG00000017425;r=12:36418017-36423059;t=ENSBTAT00000023167) |
| *Vicugna pacos* | ENSVPAP00000000328 |
| *Xenopus tropicalis* | [ENSXETP00000055228](http://www.ensembl.org/Xenopus_tropicalis/Transcript/ProteinSummary?db=core;g=ENSXETG00000026112;r=scaffold_329:930788-939774;t=ENSXETT00000055228) |

^a^See <http://www.ensembl.org/index.html>.
