# Supplementary material for: GJB2 Mutation Spectrum and Genotype-Phenotype Correlation in 1067 Han Chinese Subjects with Non-Syndromic Hearing Loss
Source: PLoS One. 2015 Jun 4;10(6):e0128691. doi: 10.1371/journal.pone.0128691 (PMC4456361; doi:10.1371/journal.pone.0128691)
Supplement: S3 Table — (DOCX) [file pone.0128691.s004.docx]

**Table S3. Genotypes of *GJB2* gene in 1067 Han Chinese subjects with hearing loss.**

| **Genotypes** | | **Number in**  **affected subjects** | **Frequency in affected subjects (%)** | **Frequency in controls (%)** | **Reported** |
| --- | --- | --- | --- | --- | --- |
| Homozygous mutation (6) | |  |  |  |  |
|  | **[c.235delC]^a^ / [c.235delC]** | 108 | 10.12 | 0.00 | Y |
|  | **[c.235delC**; c.478G>A**] / [c.235delC]** | 1 | 0.09 | 0.00 | N |
|  | **[c.235delC**; c.478G>A**] / [c.235delC**; c.478G>A**]** | 1 | 0.09 | 0.00 | N |
|  | **[c.299_300delAT] / [c.299_300delAT]** | 6 | 0.56 | 0.00 | Y |
|  | **[c.109G>A] / [c.109G>A]** | 34 | 3.19 | 0.49 | Y |
|  | [c.79G>A; **c.109G>A**;c.341A>G] **/ [c.109G>A]** | 3 | 0.28 | 0.00 | Y |
|  | **Total** | **153** | **14.34** | **0.00** |  |
| Compound heterozygous mutation (20) | |  |  |  |  |
|  | **[c.235delC] / [c.35insG]** | 2 | 0.19 | 0.00 | Y |
|  | **[c.235delC] / [c.35delG]** | 1 | 0.09 | 0.00 | Y |
|  | **[c.235delC] / [c.35G>T**;c.79G>A; c.341A>G**]** | 1 | 0.09 | 0.00 | N |
|  | **[c.235delC] / [c.127G>T**;c.79G>A; c.341A>G**]** | 1 | 0.09 | 0.00 | N |
|  | **[c.235delC] / [c.139G>T]** | 1 | 0.09 | 0.00 | Y |
|  | **[c.235delC] / [c.176_191del16]** | 7 | 0.67 | 0.00 | Y |
|  | **[c.235delC] / [c.299_300delAT]** | 19 | 1.78 | 0.00 | Y |
|  | **[c.235delC**;79G>A] **/ [c.299_300delAT]** | 1 | 0.09 | 0.00 | Y |
|  | **[c.235delC**;79G>A] **/ [c.439G>A]** | 2 | 0.19 | 0.00 | N |
|  | **[c.235delC] / [c.512_513insAACG]** | 6 | 0.56 | 0.00 | Y |
|  | **[c.299_300delAT] / [c.139G>T]** | 1 | 0.09 | 0.00 | Y |
|  | **[c.299_300delAT] / [c.176_191del16]** | 5 | 0.47 | 0.00 | Y |
|  | **[c.299_300delAT] / [c.512_513insAACG]** | 1 | 0.09 | 0.00 | Y |
|  | **[c.235delC] / [c.109G>A]** | 5 | 0.47 | 0.00 | Y |
|  | **[c.176_191del16] / [c.109G>A]** | 2 | 0.19 | 0.00 | N |
|  | **[c.283G>A] / [c.109G>A]** | 1 | 0.09 | 0.00 | N |
|  | **[c.283G>A;** c.79G>A; c.341A>G] **/ [c.109G>A]** | 1 | 0.09 | 0.00 | N |
|  | **[c.293G>C] / [c.109G>A]** | 1 | 0.09 | 0.00 | N |
|  | **[c.299_300delAT] / [c.109G>A]** | 3 | 0.28 | 0.00 | Y |
|  | **[c.427C>T] / [c.109G>A]** | 2 | 0.19 | 0.00 | N |
|  | **Total** | **63** | **5.90** | **0.00** |  |
| Heterozygous mutation (18) | |  |  |  |  |
|  | **[c.235delC] /** [+]^b^ | 21 | 1.97 | 0.00 | Y |
|  | **[c.235delC] /** [c.79G>A] | 4 | 0.37 | 0.00 | Y |
|  | **[c.235delC] /** [c.341A>G] | 1 | 0.09 | 0.00 | Y |
|  | **[c.235delC] /** [c.79G>A; c.341A>G] | 5 | 0.47 | 0.00 | Y |
|  | **[c.235delC] /** [c.79G>A;368C>A] | 1 | 0.09 | 0.00 | N |
|  | **[c.35delG] /** [c.79G>A] | 1 | 0.09 | 0.00 | N |
|  | **[c.107T>C] /** [c.79G>A; c.341A>G] | 1 | 0.09 | 0.00 | N |
|  | **[c.187G>T] /** [c.79G>A; c.341A>G] | 1 | 0.09 | 0.00 | N |
|  | **[c.176_191del16] /** [+] | 2 | 0.19 | 0.00 | Y |
|  | **[c.299_300delAT] /** [+] | 6 | 0.56 | 0.00 | Y |
|  | **[c.512_513insAACG] / [+]** | 1 | 0.09 | 0.00 | Y |
|  | **[c.512_513insAACG] /[**c.11G>A] | 1 | 0.09 | 0.00 | N |
|  | **[c.512_513insAACG] /**[c.79G>A; c.341A>G] | 2 | 0.19 | 0.00 | N |
|  | **[c.109G>A]/** [+] | 72 | 6.75 | 5.91 | Y |
|  | [c.11G>A] **/ [c.109G>A]** | 1 | 0.09 | 0.00 | Y |
|  | [c.79G>A] **/ [c.109G>A]** | 9 | 0.84 | 0.49 | Y |
|  | **[c.109G>A]/** [c.341A>G] | 1 | 0.09 | 0.00 | Y |
|  | [c.79G>A; c.341A>G] **/ [c.109G>A]** | 27 | 2.53 | 2.46 | Y |
|  | **Total** | **157** | **14.71** | **0.00** |  |
| Polymorphism (21) | |  |  |  |  |
|  | [c.11G>A] **/** [+] | 4 | 0.37 | 0 | Y |
|  | [c.79G>A] **/** [c.79G>A] | 4 | 0.37 | 2.46 | Y |
|  | [c.79G>A] **/** [+] | 67 | 6.28 | 7.39 | Y |
|  | [c.341A>G] **/** [c.341A>G] | 1 | 0.09 | 0.00 | Y |
|  | [c.341A>G] **/** [+] | 2 | 0.19 | 0.00 | Y |
|  | [c.444C>T] **/** [+] | 1 | 0.09 | 0.00 | N |
|  | [c.608T>C] **/** [+] | 2 | 0.19 | 0.00 | Y |
|  | [c.79G>A; c.341A>G] **/** [c.79G>A; c.341A>G] | 41 | 3.84 | 1.97 | Y |
|  | [c.79G>A] **/** [c.79G>A; c.341A>G] | 27 | 2.53 | 4.43 | Y |
|  | [c.79G>A; c.341A>G] **/** [c.341A>G] | 1 | 0.09 | 1.48 | Y |
|  | [c.79G>A; c.341A>G] **/** [+] | 237 | 22.21 | 16.26 | Y |
|  | [c.79G>A] **/** [c.368C>A] | 12 | 1.12 | 0.00 | Y |
|  | [c.79G>A] **/** [c.608T>C] | 1 | 0.09 | 0.00 | Y |
|  | [c.11G>A] **/** [c.79G>A; c.341A>G] | 1 | 0.09 | 0.00 | N |
|  | [c.79G>A; c.341A>G] **/** [c.81C>T] | 1 | 0.09 | 0.00 | N |
|  | [c.478G>A] **/** [c.79G>A] | 1 | 0.09 | 0.00 | N |
|  | [c.478G>A] **/** [c.79G>A; c.341A>G] | 1 | 0.09 | 0.00 | N |
|  | [c.478G>A; c.79G>A] **/** [c.79G>A; c.341A>G] | 1 | 0.09 | 0.00 | N |
|  | [c.79G>A; c.341A>G] **/** [c.79G>A;c.368C>A] | 4 | 0.37 | 0.00 | Y |
|  | [c.88A>G; c.79G>A;c.341A>G] **/** [c.88A>G;c.79G>A] | 1 | 0.09 | 0.00 | N |
|  | [c.88A>G;c.79G>A;c.341A>G] **/** [c.79G>A;c.341A>G] | 1 | 0.09 | 0.00 | N |
|  | **Total** | **411** | **38.52** | **43.34** |  |
|  | **[+] / [+]** | 283 | 26.52 | 56.65 | Y |
| TOTAL | | **1067** | **100.00** | **100.00** |  |

^a^The pathogenic mutations were in bold.

^b^+ denoted as Wild Type.
